# Is Epstein-Barr Virus Infection Associated With Thyroid Tumorigenesis?—A Southern China Cohort Study

**DOI:** 10.3389/fonc.2019.00312

**Published:** 2019-04-26

**Authors:** Shi-Tong Yu, Jun-Na Ge, Rui-Chen Li, Zhi-Gang Wei, Bai-Hui Sun, Yu-Ming Jiang, Jing-Yi Luo, Hao Liu, Shang-Tong Lei

**Affiliations:** ^1^Department of General Surgery, Nanfang Hospital, Southern Medical University, Guangzhou, China; ^2^Department of Radiation Oncology, Eye & ENT Hospital, Fudan University, Shanghai, China; ^3^State Key Laboratory of Ophthalmology, Zhongshan Ophthalmic Center, Sun Yat-sen University, Guangzhou, China

**Keywords:** thyroid cancer (TC), Epstein-Barr virus (EBV) infection, EBER = EBV-encoded small RNA, EBV-specific capsid antigen (VCA/IgA), EBV-specific early antigen (EA/IgA), Southern China

## Abstract

**Background:** Epstein-Barr virus (EBV) is associated with many epithelial malignancies. A few reports on the association between EBV and thyroid tumorigenesis have been investigated. However, the conclusion is highly contradictory. We aimed to explore the role of EBV in thyroid nodule development and its clinical significance in a cohort from southern China.

**Method:** We conducted a retrospective data abstraction study of patients who underwent thyroidectomy between December 2017 and June 2018. We retrospectively analyzed the clinicopathological parameters and EBV infection status (serological antibodies and *in situ* hybridization).

**Result:** The cohort comprised 384 patients with newly diagnosed thyroid diseases, including 261 papillary thyroid carcinomas, 87 nodular goiters, 21 follicular adenomas, 12 follicular thyroid carcinomas, and 3 medullary thyroid carcinomas. Forty-two (10.9%) patients were identified as being serological antibody positive. However, there was no association between the clinicopathological parameters and serological antibody positivity. Additionally, none of the patients showed EBER expression in thyroid normal/cancer cell nuclei in *in situ* hybridization.

**Conclusion:** In this study, no correlation between EBV and thyroid diseases was found in a cohort from southern China.

## Introduction

Epstein-Barr virus (EBV) is a well-known human tumor virus with a very high prevalence in the population, especially for children and youth. EBV serum (IgG) is positive in an estimated 95% of the world's population (1). EBV infection is associated with epithelial and lymphoid malignancies, including nasopharyngeal carcinoma (NPC), gastric cancer, Hodgkin's lymphoma and Burkitt's lymphoma (2, 3). Although EBV has B-lymphocyte tropism, it can also infect T lymphocytes, myocytes, and epithelial cells in the oropharynx and stomach (4). Once EBV infects a host cell, it starts to induce a lytic or latent infection with diverse genes expressed. EBV nuclear antigens (EBNA 1, 2, 3A, 3B, 3C, and LP), the latent membrane proteins (LMP 1, 2A, and 2B) and two small noncoding RNAs (EBV-coded small RNA, EBER-1, and EBER-2) are expressed during the infection (3). These genes collaborate to induce tumorigenesis by causing systematic inflammation, suppressing the antitumoral immune system, and preventing anoikis.

Whether EBV infects the thyroid gland remains controversial. To date, only a handful of reports on the association between EBV and thyroid tumorigenesis have been investigated. Stamatiou et al. (5) summarized publications regarding the EBV detection rate in thyroid cancer specimens from 2001 to 2015. The conclusion was inconclusive because the results were highly contradictory, ranging from negative to 100% positive for EBV infection.

Therefore, in the current study, we explored the role of EBV in thyroid nodule development and its clinical significance in a cohort from southern China.

## Methods

### Patient Selection and Sample Collection

A total of 384 patients, including 261 with papillary thyroid carcinomas, 87 with nodular goiters, 21 with follicular adenomas, 12 with follicular thyroid carcinomas, and 3 with medullary thyroid carcinomas, who underwent thyroidectomy between December 2017 and June 2018 at the Department of General Surgery, Nanfang Hospital, Southern Medical University, were identified as being eligible for the study. All the data were extracted from the database of the Department of General Surgery, Nanfang Hospital, Southern Medical University. The patients included in the study met the following criteria: (1) primary thyroid neoplasms (including thyroid cancer, nodular goiter and follicular adenoma) confirmed by post-operative pathological results, (2) no history of thyroid/neck surgery, (3) no previous diagnosis of nasopharyngeal carcinoma (NPC) or other EBV-related disease, (4) no history of neck radiotherapy, (5) exclusion of cervical metastatic cancer, parathyroid neoplasms and Graves' disease, and (6) sufficient medical history. All patients were invited to donate a 5-mL blood sample for storage when they received preoperative examinations in our department. Blood samples were allowed a maximum of 6 h at room temperature before serologic analysis. The serum samples were then separated and divided into 4 tubes. All the serum samples were stored at −80°C at the Department of General Surgery, Nanfang Hospital, Southern Medical University. All the tissues samples were formalin fixed and paraffin embedded (FFPE) and then were cut into 4-μm-thick sections. All the clinicopathological parameters were recorded and evaluated according to the criteria of the American Joint Committee on Cancer (AJCC, 8th edition). This study was approved by the ethical committee of Nanfang Hospital, Southern Medical University. Informed consents were obtained from all involved patients when they were admitted to the hospital to provide their test results/specimens for future medical research.

### *In situ* Hybridization

The EBER-ISH (*in situ* hybridization) test is the most commonly employed method and is regarded as the gold standard to diagnose EBV-infected diseases. We evaluated the presence of EBV in thyroid cells by *in situ* hybridization (ISH) analysis using EBV-encoded small RNA (EBER) probes, PNA probe/FITC (code Y5200), and the PNA ISH detection kit (code K5201) (Dako, Denmark) on the FFPE samples. The protocol was performed according to the manufacturer instructions. Briefly, the slides were baked at 60°C for 2 h and then were deparaffinized using a standard protocol. Deparaffinized slides were boiled for 20 min in pH 6.0 citric buffer for antigen retrieval. The slides were further permeabilized by Protease III treatment at room temperature for up to 10 min. The slides were then hybridized at 40°C for 2 h, followed by amplification and detection by adding Amp 1–4. The primary antibody incubation time at 4°C varied from 12 to 16 h. The substrate was incubated for 60 min, followed by counterstaining with eosin and mounting using Aquamount (Dako). EBER expression was graded from negative (–) to slight (+), moderate or intensive (+++), where most cells express EBER-RNA. The procedure was conducted by an experienced pathologist. Additionally, ISH was performed individually using 3 slides in each patient. Previously known cases of EBV-positive Hodgkin's lymphoma and NPC were used as positive controls. Because there is no established cutoff for EBER in solid malignancies other than NPC or gastric cancer (6), we referred this cutoff (>5%) of dark-brown staining of the tumor nucleus as positive for EBER transcript expression.

### Serologic Antibody Analysis

Serologic antibody analysis was conducted in all patients using EBV-specific capsid antigen (VCA/IgA) antibodies and EBV-specific early antigen (EA/IgA) antibodies. Serological analysis was performed on July 2018 at the Department of Laboratory Medicine, Nanfang hospital, Southern Medical University because VCA-IgA and EA-IgA were routinely tested for screening NPC in Southern China institutions (7–9). Validation of the serological data was existing results from patients who underwent these two tests at our hospital between December 2017 and June 2018. Positive groups were patients with NPC, and healthy controls were the normal population from the medical examination center. Their ages and sex were matched with those in the thyroid disease cohort (1:1). EBV-specific VCA/IgA antibodies and EA/IgA antibodies were measured by ELISA (Euroimmun, Lubeck, Germany). The levels of these seromarkers were measured photometrically, according to the manufacturers' instructions. Additionally, these results were standardized by calculating the ratio of the optical density (OD) of the sample over that of the reference control (rOD). If the rOD value was >1, the sample was regarded as positive (10, 11). Either VCA/IgA or EA/IgA was positive, and the patients were regarded as serological antibody positive.

### Statistical Analysis

All continuous variables were expressed as medians (Percentile 25, Percentile 75). Statistical analysis was conducted using SPSS 22.0 (SPSS, Inc, Chicago, IL, USA). To explore the relationship between EBV and the clinical pathological features in PTC, such as gender, age, and tumor size, chi-squared test and Fisher's exact test were used as appropriate; *p* < 0.05 considered as statistically significant.

## Results

**Figure 1:**
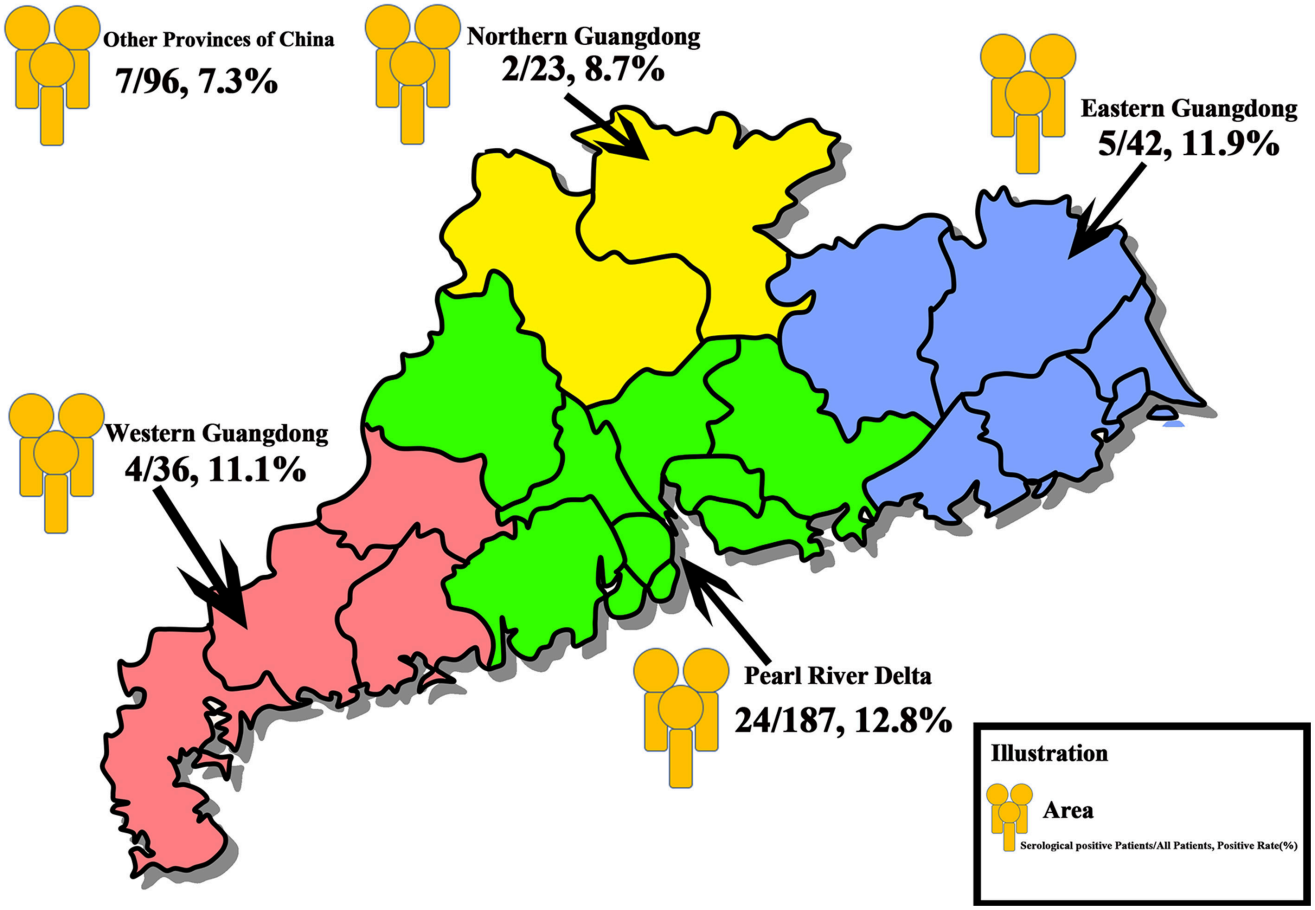
Overall distribution of the study population. In total, 384 patients with thyroid disease who underwent thyroidectomy were recruited. A total of 187 patients were from the Pearl River Delta (Green Area), 24 (12.8%) patients were positive for serological antibodies test, 42 patients were from the Eastern Guangdong (Blue Area), 5 (11.9%) patients were serological positive, 36 patients and 23 patients were from the Western (Red Area) and the Northern (Yellow Area) Guangdong, with a positive rate of 11.1% (4 patients) and 8.7% (2 patients), respectively. Ninety-six patients were from other provinces of China, and 7 patients (7.3%) were serologically positive.

The clinicopathological features are presented in Table 1. The median age of the included patients was 45 years (40, 61). The median number of resected lymph nodes in Level VI (for those with central neck dissection) and Level II-Vb (for those with lateral neck dissection) were 11.5 (7, 15) and 40 (25.75, 58.75), respectively. Two hundred eighty eight patients came from Guangdong Province (Southern China), and 96 patients came from other provinces of China (Figure 1).

**Table 1:** Baseline characteristics of all thyroid neoplams patients.

**Characteristics**	***n***	**%**
Gender	All = 384	
Male	153	39.8
Female	231	60.2
Pathology		
Nodular goiter	87	22.7
Follicular adenoma	21	5.5
Papillary thyroid carcinoma	261	68
Follicular thyroid carcinoma	12	3.1
Medullary thyroid carcinoma	3	0.8
VCA/IgA positivity	29	7.6
EA/IgA positivity	19	4.9
Serological positivity	42	10.9
EBER positivity	0	0
T stage	Thyroid carcinoma = 276	
1	216	78.3
2	30	10.9
3	21	7.6
4	9	3.3
N stage		
0	102	37
1a	117	42.4
1b	57	20.6
M stage		
0	270	97.8
1	6	2.2
AJCC stage		
1	232	84.1
2	26	9.42
3	12	4.3
4	6	2.2
Strap muscles invasion	24	8.7
Multifocality	57	20.7
Bilaterial	36	13
Hashimoto's thyroiditis	36	13

### Serological Antibodies

The VCA/IgA and EA/IgA antibodies were tested in all 384 patients with thyroid nodules as well as the population of positive/normal controls. There were 42 (10.9%) patients who were either VCA/IgA antibody or EA/IgA antibody positive or positive for both antibodies; therefore, these patients were designated as the serological antibody positive group. Comparing all clinicopathological features between the serological antibody groups, none of these parameters with statistical significance were identified (Supplementary Tables 1, 2). The number of patients with available serological results was 857 and 6,923 for NPC (positive control) and normal population (healthy control). After matched ages and sex, 384 NPC patients (positive control) and 384 healthy controls (normal population) were compared with those of the thyroid cohort (Supplementary Table 3). There were 311 (81.0%) patients who were serological positive in positive controls, and 40 (10.4%) patients with positive serological tests in the normal population.

### *In situ* hybridization

**Figure 2:**
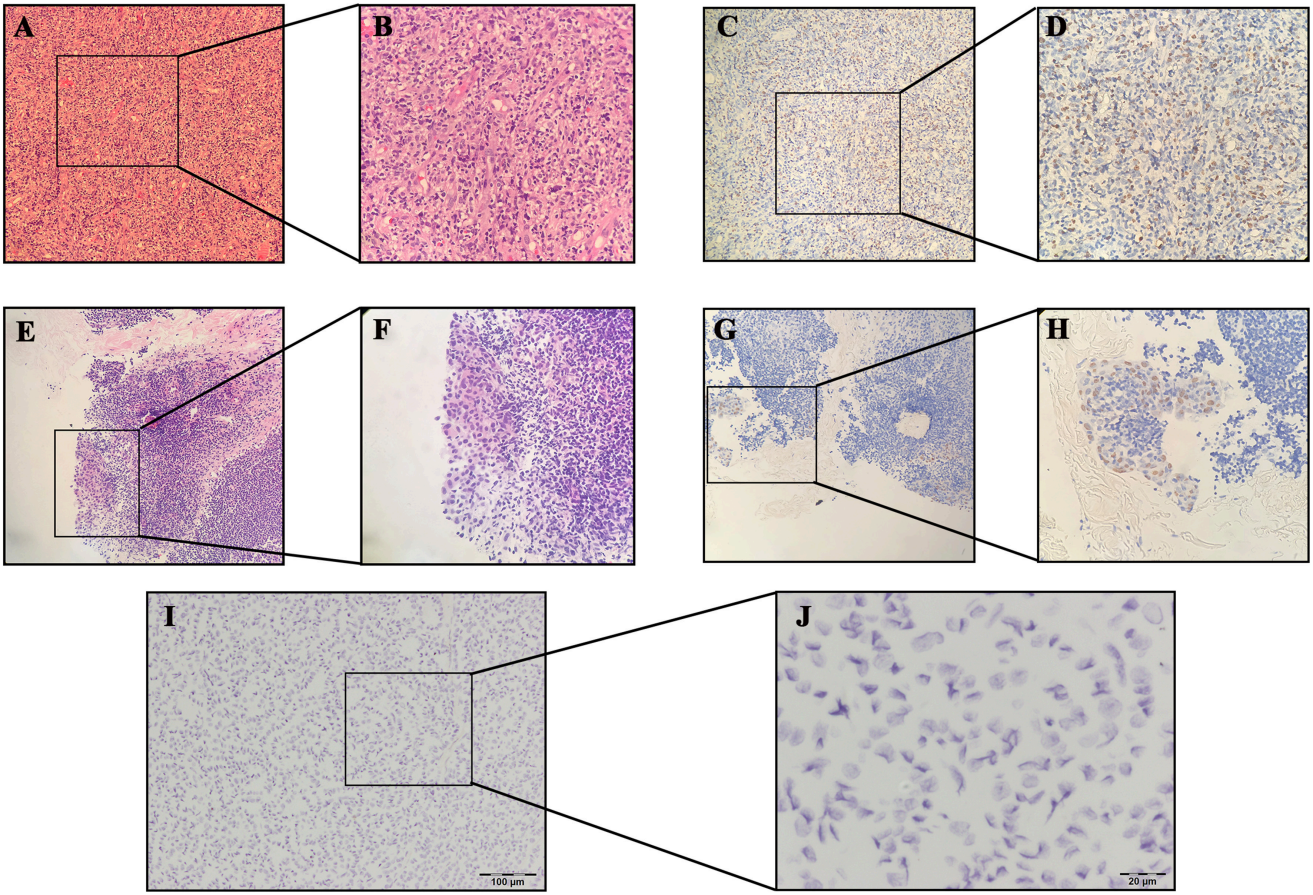
Results of hematoxylin-eosin (HE) staining of Hodgkin's lymphoma: **(A)** 10×; **(B)** 40×; Results of EBER-*in situ* hybridization (ISH) of Hodgkin's lymphoma: **(C)** 10×; **(D)** 40×; Results of HE staining of nasopharyngeal carcinoma (NPC): **(E)** 10×; **(F):** 40×; Results of EBER-ISH of NPC: **(G)** 10×; **(H)** 40×; Results of EBER-ISH of papillary thyroid carcinoma: **(I)** 10×; **(J)** 40×.

None of the samples analyzed by ISH showed EBER expression in thyroid normal/cancer cell nuclei, even those with positive results in serological antibody analysis (Figure 2).

## Discussion

EBV has been revealed to be associated with the development of many cancers, such as gastric cancer, NPC, and Hodgkin's lymphoma (3). Chronic inflammation induced by EBV infection may play a significant role in the progression of cancer (4). However, the relationship between thyroid tumorigeneses and EBV has not been fully elucidated with conflicting results. The preliminary investigation of EBV in thyroid lymphoma was inspired by EBV persistently infecting B lymphocytes, contributing to lymphoma formation. In 2003, Shimakage et al. first reported EBV infection in other types of thyroid malignancies with a Japanese cohort (12). Shimakage et al. (12) explored the potential involvement of EBV expression in the progression of thyroid cancer by examining different thyroid neoplasm specimens, ranging from PTC to anaplastic thyroid carcinoma (ATC). The specimens were subjected to PCR, RT-PCR, ISH and indirect immunofluorescence staining. The results showed that mRNA and protein were positive for all carcinoma specimens and their expression was prominent in ATCs. For benign nodules, they showed no signal or very few signals during ISH. A similar result was conducted by Moghoofei et al. (13). The authors determined EBV infection by qRT-PCR and revealed that the EBV detection rate in PTC was similar to that of the healthy control. However, EBV positivity was associated with the tumor stage. Additionally, based on the PCR result, several inflammatory factors, such as Survivin, CD44, NF-kappaB, and IL-6, were higher expressed in the EBV-positive groups, and the mRNA expression of EBER1 and EBER2 was higher in thyroid tumor group. The reports from Almeida et al. (14) and Homayouni et al. (15) conducted similar results based on PCR and the ISH test in Brazilian and Iranian populations, respectively.

However, negative results for the association between thyroid tumors and EBV infection have been obtained. Despite a few infiltrating lymphoid cells in 1 (2.2%) of the specimens, none of the 45 PTCs were positive for the EBER-ISH test in a Japanese cohort (16). Bychkov et al. (17) reported that 1 of 20 thyroid cancer tissues contained single EBER-positive inflammatory cells. Cancer cells and normal thyroid tissues were consistently negative for ISH. Additionally, Tsai et al. (18) reported a negative association between benign tumors and EBV infection using ISH or PCR or Southern hybridization in a Taiwanese population.

To the best of our knowledge, this is the first study regarding EBV and thyroid neoplasms based on serological antibodies and ISH analyses in a cohort from the southern part of China. Similar to other southeast Asia neighbors, EBV is highly prevalent in southern China. Additionally, NPC, which has a closer relationship with EBV infection, is more endemic than in any part of the world, especially in Guangdong Province and Hong Kong (19). VCA/IgA and EA/IgA antibodies could reflect the status of recent viral infection and, therefore, are widely used biomarkers for screening NPC in the southern China population (8). In the current study, the positivity rate of serological antibody analysis was 10.9%, which is similar to that in previous national population-based studies conducted in the 1970s (7–9). However, there is no meaningful results between the clinicopathological parameters and VCA/IgA or EA/IgA antibodies. Furthermore, we failed to detect EBER signals in thyroid cancer cells or normal thyroid cells based on the ISH test. Interestingly, two PTC patients who were serological test positive with concurrent NPC were enrolled in this cohort. These two patients had pathologically confirmed NPC by preoperative fiber-laryngoscopy accidentally. They all received radiotherapy for NPC after thyroidectomy and were disease-free at the last follow up. However, we failed to detect the EBER signal in thyroid cancer cells, although the NPC specimens showed nasopharyngeal carcinoma cells that were positive for ISH. Our data indicated that the EBV detection rate in thyroid tumor samples from the southern China population is extremely low, consistent with previous EBER-based reports but differed from PCR-based tests. Several reasons may explain our findings. First, there is difference in populations and geography among different studies. Second, diversity in EBV detection techniques and their interpretation may cause conflicting results. Bychokov A believed that not only viral load assays (PCR or qPCR) but histochemical assays (ISH, immunofluorescence and immunohistochemistry) should be implied to ensure the precise tissue detection of EBV (17). A false-positive result may be received because any tissue containing B lymphocytes may have traces of EBV DNA (20). Additionally, there is no standard for the interpretation of EBV positivity for the ISH EBER test in thyroid tumors. Previous studies with positive results, which set a cutoff of few (<5%) EBER-positive cancer cells and even a single EBER-positive cancer cell are questionable because, in hematological malignancy, <1% of the EBER-positive background lymphocytes are usually regarded as negative during evaluation (20).

Indeed, in the current study, there are many limitations. First, the retrospective study nature may cause inevitable bias. Second, serological antibody analysis only involved VCA/IgA and EA/IgA antibodies and may be insufficient for EBV viral load evaluation; more comprehensive assays should be included in further studies. Third, only the EBER-based ISH test was included in the current study. Other viral detection methodologies, such as the LMP-1 immunohistochemical test, could be implied to better evaluate EBV infection in a future study.

## Conclusion

We found no correlation between EBV and thyroid diseases in our study based on the current evidence. Future studies are warranted to reveal the significance of EBV in thyroid tumor developments.

## Ethics Statement

This study was approved by the ethics committee of Nanfang Hospital, Southern Medical University. Informed consents were obtained from all enrolled patients.

## Author Contributions

S-TL and HL designed the study. S-TY, J-NG, and R-CL conducted the analysis. S-TY and R-CL drafted the manuscript. Z-GW, B-HS, Y-MJ, and J-YL participated in result interpretation. All authors have read the manuscript and approved for publication.

### Conflict of Interest Statement

The authors declare that the research was conducted in the absence of any commercial or financial relationships that could be construed as a potential conflict of interest.
