# Supplementary material for: Is Epstein-Barr Virus Infection Associated With Thyroid Tumorigenesis?—A Southern China Cohort Study
Source: Front Oncol. 2019 Apr 26;9:312. doi: 10.3389/fonc.2019.00312 (PMC6524691; doi:10.3389/fonc.2019.00312)
Supplement: Supplementary file 1 [file Data_Sheet_1.pdf]

Supplementary Table 1

The correlation between serological analysis and characteristics in all patients

Characteristics		Serological group		P
		Negative (n= 342)	Positive (n= 42)	
Age				0.72
	<55	253	30	
	≥55	89	12	
Gender				0.51
	Male	138	15	
	Female	204	27	
Hashimoto's thyroiditis		30	6	0.39
Pathology				0.21
	Benign	100	8	
	Maglinancy	242	34	

Supplementary Table 2

The correlation between serological analysis and characteristics in TC patients

Characteristics		Serological group		P
		Negative (n= 242)	Positive (n= 34)	
Strap muscle invasion				0.99
	Yes	21	3	
	No	221	31	
Multifocality				0.37
	Yes	48	9	
	No	194	25	
Bilaterial				0.16
	Yes	29	7	
	No	213	27	
T stage				0.38
	1	190	26	
	2	24	6	
	3	19	2	
	4	9	0	
N stage				0.23
	0	85	17	
	1a	105	12	
	1b	52	5	
M stage				0.16
	0	238	32	
	1	4	2	
AJCC stage				0.47
	I+II	227	31	
	III+IV	15	3	

Abbreviation: TC, thyroid cancer

Supplementary Table 3

Serological analysis in three different groups

Characteristics	Nasopharyngeal carcinoma<br>n=384 (%)	Normal population<br>n=384 (%)	Thyroid cohort<br>n=384 (%)
Age ≥55	100 (26.0%)	100 (26.0%)	100 (26.0%)
Sex (male/female)	153:231	153:231	153:231
VCA-IgA			
Negative	84 (21.9%)	344 (89.6%)	340 (92.4%)
Positive	300 (78.1%)	40 (10.4%)	29 (7.6%)
EA-IgA			
Negative	250 (65.1%)	372 (96.8%)	365 (95.1%)
Positive	134 (34.9%)	12 (3.2%)	19 (4.9%)
Serological results *			
Negative	73 (19.0%)	344 (89.6%)	342 (89.1%)
Positive	311 (81.0%)	40 (10.4%)	42 (10.9%)

* VCA-IgA and EA-IgA may both positive in the same patients
